# Dose tolerance limits and dose volume histogram evaluation for stereotactic body radiotherapy

**DOI:** 10.1120/jacmp.v12i2.3368

**Published:** 2011-02-08

**Authors:** Jimm Grimm, Tamara LaCouture, Raymond Croce, Inhwan Yeo, Yunping Zhu, Jinyu Xue

**Affiliations:** ^1^ Department of Radiation Oncology Cooper University Hospital, One Cooper Plaza Camden NJ 08103 USA

**Keywords:** dose tolerance limits, adverse events, stereotactic body radiotherapy, radiosurgery, DVH evaluation

## Abstract

Almost 20 years ago, Emami et al. presented a comprehensive set of dose tolerance limits for normal tissue organs to therapeutic radiation, which has proven essential to the field of radiation oncology. The paradigm of stereotactic body radiotherapy (SBRT) has dramatically different dosing schemes but, to date, there has still been no comprehensive set of SBRT normal organ dose tolerance limits. As an initial step toward that goal, we performed an extensive review of the literature to compare dose limits utilized and reported in existing publications. The impact on dose tolerance limits of some key aspects of the methods and materials of the various authors is discussed. We have organized a table of 500 dose tolerance limits of normal structures for SBRT. We still observed several dose limits that are unknown or not validated. Data for SBRT dose tolerance limits are still preliminary and further clinical trials and validation are required. This manuscript presents an extensive collection of normal organ dose tolerance limits to facilitate both clinical application and further research.

PACS numbers: 87.53.Ly, 87.55.dk

## I. INTRODUCTION

In a recent journal article, Papiez and Timmerman^(1)^ identified the most important area in which stereotactic body radiotherapy (SBRT) needs to mature to reach its full potential: “The main obstacle for safe application of the SBRT treatment technique is the unavailability of data that allow unambiguous determination of the parameters for fractionation schemes and dose prescriptions.” It is the aim of this review to present updated dose tolerance limits to all critical structures for SBRT fractionation schemes, and to extend the historical data as much as possible, based on recent publications. We consider SBRT to be small, focused, stereotactically accurate radiation treatments with one to five fractions^(2)^ delivered anywhere in the body.

This manuscript represents Phase I of a three‐phase project to determine SBRT dose tolerance limits. This first phase is to assemble the range of SBRT dose tolerance limits that have been used clinically and organize them into a format suitable for future research. Some of these dose limits are from randomized trials that have already completed accrual like RTOG 0618, but even in most of those instances, the follow‐up data have not yet been published. At the other extreme, some of the published dose tolerance limits are only from a single case study, and some report doses that caused adverse events and should be avoided. Our Phase II effort is to analyze these dose tolerance limits, consolidate them into expert‐opinion “high‐risk” and “low‐risk” categories, and provide dose tolerance limits in a unified framework from one to five fractions. We are working with many other researchers on the Phase II effort, but we feel that the content of these Phase I dose limits should already be quite useful for research. However, few of these Phase I limits are based on clinical outcome data and these dose tolerance limits have not been validated, so clinical use of them could be hazardous unless the caveats from each publication are heeded. The Phase III effort will be a statistical analysis of long‐term multi‐institutional follow‐up data, to determine the real dose tolerance limits.

The literature already contains a wealth of information regarding the various tumor types treated, as well as the rationale and strategy for defining and delivering radiosurgical treatments. In this manuscript, we avoid what is readily available elsewhere and refer the reader to the literature. Instead, we attempt to aggregate and organize all of the currently‐published dose tolerance limits into a single manuscript, and limit the discussion to some of the nuances of those dose tolerance limits. Even with such limited focus, it is still not possible to explain all details of the dose tolerance limits fully, and the reader is still referred to the numerous references to thoroughly understand the background of each dose tolerance limit.

After 20 years of RTOG trials, conventional radiation therapy had progressed to the point where Emami et al.^(3)^ could, with relative certainty, pose the dose tolerance limits in terms of 5% or 50% chance of a specified adverse event occurring within five years. In contrast, the published follow‐up data for SBRT are inadequate to reliably determine probability of adverse events. In the meantime, it is important to have a review such as this manuscript that extensively summarizes the dose limits that are currently being used, and to summarize some of the issues regarding those limits.

The simplicity of the Emami limits is that all prescriptions were in 1.8 or 2 Gy fractions. In contrast, SBRT prescription schemes typically range from 5 Gy per fraction up to 20 Gy per fraction or more. In this new radiation delivery paradigm, normal organ dose tolerance limits and the dose‐volume response of the tumors depend strongly on the number of fractions used and the dose per fraction. In order to completely fulfill the need that Papiez and Timmerman recommended,^(1)^ it will take years of carefully planned randomized trials to truly determine the optimum prescriptions – clearly beyond the scope of any single manuscript. Until these experiences can be generated, this work attempts to summarize the available dose tolerance limits from the literature and establish some boundaries within which doses can be prescribed.

Several review articles have recently presented a wealth of dose tolerance limits in convenient tabular form.^(^ ^4^ ^,^ ^5^ ^,^ ^6^ ^,^ ^7^ ^,^ ^8^ ^)^ As useful as they are, each article only reveals a few dozen dose limits, and the clinical user soon realizes that many needed limits are still missing. This manuscript presents 500 dose tolerance limits, for a broader range of anatomical structures, for relatively more complete coverage of the SBRT fractionation schemes, and to show the variation of limits among many of the leading institutions. In SBRT, the goal is frequently to deliver the highest possible dose to the tumor; hence, we often find ourselves prescribing to meet the dose limits rather than prescribing to the ideal prescription the physician desires. This means a solid understanding of dose tolerance limits is of paramount importance.

Treating an area that had prior radiation is far more complex than the initial treatment. Some of the references address the issue, although most do not. Retreatments are beyond the scope of this manuscript.

The equation for biological effective dose (BED) provides a simple and straightforward way to compare doses from different fractionation schemes.^(^ ^9^ ^–^ ^13^ ^)^ It would be possible to simply convert the Emami dose limits to any fractionation scheme using this equation, as was done in.^(14)^ Although these tables are a very important reference tool, they leave out an enormous amount of detail. The response to radiation delivered in one to five fractions could be dramatically different from the response in 25 to 40 fractions – beyond the ability of a simple equation to predict. This type of theoretical analysis is helpful to form the basis for clinical investigations, but by itself has no clinical validation.

Many critics of the work regarding SBRT dose tolerance limits point out limitations that have not even been fully resolved for conventional fractionation. For example, the lower lobes of the lungs may be more sensitive to radiation pneumonitis than the upper lobes. Another example is the variable time period in which the treatments were delivered – some are daily treatments, others are for every‐other‐day, or weekly or bi‐weekly treatments. These are all important ongoing research topics, but since these effects are still somewhat unknown for conventional radiation, it cannot be expected to be known from the start for SBRT.

We cannot stress emphatically enough that we are not claiming any of the dose limits presented in this work are safe, and we are not claiming that any of them actually are the maximal attainable safe dose. Many clinical trials are still needed to determine the best limits. It is the responsibility of each physician to determine which dose limits are appropriate for their patients.

## II. MATERIALS AND METHODS

In a literature review, there is a wide variation of the methods and materials of each cited reference, and it is important for the reader to understand how that variability can affect the presented results. The data presented herein also have a wide variation in validation and reliability. At one extreme, some references are large‐scale randomized RTOG trials, and at the other extreme, some of the references are a case study of just a single patient. In nearly all references, the follow‐up period is inadequate to assess late effects, but it is still essential to initiate reviews such as this early, to at least see what dose limits other researchers believe are appropriate.

After searching through hundreds of journal articles, we are still missing SBRT dose tolerance limits for many critical structures such as gallbladder, oral mucosa, ovaries, pancreas, parotid, pituitary, spleen, testes, thyroid, ureter and vagina. There are numerous publications discussing irradiating some of these critical structures when they harbor a malignancy – although it is more challenging to find publications that present dose tolerance limits required to maintain normal function of these organs when they are not the target.

These are not universal dose tolerance limits, and they must be used with an understanding of the treatment scenario for which they were initially developed. For example, dose tolerance limits from the RTOG 7631 trial for palliative whole brain^(15)^ should not generally be applied to curative cases for children. It is not feasible to enumerate all such possible caveats, and it is the responsibility of the clinical users to study the references and carefully consider which dose limits are appropriate for each patient.

We do not address the issue of medications that could help the patient become more resistant to the effects of radiation, or the issue of medications that could make them more sensitive. For example, if a publication used Ethyol, Zofran, Zantac, Palifermin, Decadron, or some other medication, they could potentially achieve higher dose tolerance limits, but to use those limits safely, the same medications would need to be administered. Conversely, the effects of chemotherapy, molecular targeted agents, Beαr, Zevalin, or any other concurrent therapy could potentially make patients more susceptible to complications from radiation, so the dose tolerance limits may need to be lowered in those cases. Many of these effects are known for conventional fractionation schemes, but to fully understand interactions with the extreme hypofractionation schemes of SBRT, many new clinical trials will likely be needed.

## III. RESULTS

We report all references that we found to each dose limit in Table 1, regardless of whether each author actually used the dose tolerance limit or not. Some of the higher dose limits of one author might be discussed by other authors who might not actually feel comfortable using a dose that high. It is important to realize that a large number of references for a particular dose tolerance limit could indicate that it is controversial, rather than indicating that it is commonly used.

**Table 1:** SBRT dose tolerance limits.

*Organ*	# *fx*	*Vol. cc*	*Vol.* %	*Vol. Limit (Gy)*	*Max Limit (Gy)*	*Refs.*	#AE≥G3	# *pts rx this dose*	# *pts in study*	*Notes*
	1	0.035		37		24				RTOG 0631, Limit is for ‘Great Vessels'
	1				37	8,25				RTOG 0915, Limit is for ‘Great Vessels'
	1	10		31		8,24,25				RTOG 0631&0915, Limit is for ‘Great Vessels'
	3				45	8				Limit is for ‘Great Vessels'
	3	10		39		8				Limit is for ‘Great Vessels'
	3	5		21		26				Limit is for Aorta
Aorta and Major Vessels	4			50	27				
	4				49	25				RTOG 0915, Limit is for ‘Great Vessels'
	4	10		43		25				RTOG 0915, Limit is for ‘Great Vessels'
	4	1		40		27,28				Limit is for ‘Major Vessels'
	4	10		35		27,28				Limit is for ‘Major Vessels'
	4				47.2	29,30				Limit is for Pulmonary artery
	5				53	8				Limit is for ‘Great Vessels'
	5				52.5	31				RTOG 0813, QOD, Limit is for ‘Great Vessels'
	5	10		47		8,31				RTOG 0813, QOD, Limit is for ‘Great Vessels'
Area Post‐rema	1				6.2	32,33				Use anti‐nausea medication
	1				22	8				Limit is for bladder wall
	1	15		8.7		8				Limit is for bladder wall
	1				8	34				
	2	1		14.25		35				RTOG 0321
	3				30	8				Limit is for bladder wall
	3	15		15		8				Limit is for bladder wall
	3				25	36				RTOG 9708, after 45Gy conventional
	4		10%	41.8		37				
Bladder	4				45.6	37,38				
	4				36	39				
	5				38	8				Limit is for bladder wall
	5	5		37.5		40				
	5	10		37		41				
	5	15		18.3		8				Limit is for bladder wall
	5				24	42				RTOG 0116, after 45Gy conventional
	5				23	43				RTOG 0417, after 45Gy conventional
	5				23	44				RTOG 0118, after 45Gy/25fx, Section 6.2.3.8
	5				21	44				RTOG 0118, after 45Gy/25fx, Appendix VIII
	1	10		14		8				
Bone: Femoral head	3	10		21.9		8				
	5	10		30		8				
	1				30	25				RTOG 0915
	1	1		22		25				RTOG 0915
	3				76.4	45	**1**		**38**	25 months after SBRT
	3	1.4		60		46	**17**		**60**	17 pain, 5 fractures
	3	2		49.8		47				ML estimate of 50% risk
	3	2.9		46.4		47	**7**	**3.5**	**33**	ML estimate of 50% risk
	3	5.5		36.4		47	**7**	**3.5**	**33**	ML estimate of 50% risk
Bone: Rib Cage	3	2.3		50		46	**17**		**60**	17 pain, 5 fractures
	3	30		30		46	**17**		**60**	17 pain, 5 fractures
	3	2		27.2		47				ML estimate of 5% risk
	4				40	25				RTOG 0915
	4	10		35		28	**3**		**27**	
	4	1		32		25				RTOG 0915
	5	1.4		60			46	**17**	**60**	17 pain, 5 fractures
	5	2.3		50			46	**17**	**60**	17 pain, 5 fractures
	5	30		30			46	**17**	**60**	17 pain, 5 fractures
	6				54	48				
Bone: TMJ	5				20	49				Limit is for mandible, QOD, re‐treat
	1			5%	20		50	**1**	**77**	G4 small bowel perforation
	1				21		50	**1**	**77**	G4 small bowel perforation
	1		5%	12.3		18				
	1				8	34				
Bowel	3	5		21		26				
	3				21	51				
	4				28.5	37				
	5	1		30		41				
	5	1		25		52				
	1	20		14.3		24				RTOG 0631
	1	0.035		18.4		24				RTOG 0631
Bowel: Colon	1				22	8				
	1	20		11		8				
	3				30	8				
	3	20		20.4		8				
	4				28.5	37				
Bowel: Colon (cont'd.)	5				38	8				
	5	1		30		41				Limit is for sigmoid colon
	5	20		25		8				
	5				21	44				RTOG 0118, after 45Gy conventional
	1		5%	22.5		18,39,50,53	**2**		**16**	After 4–10 months, G3‐4 ulceration encountered
	1	5%		22.5		50	**1**		**77**	
	1				16	8				
	1	5		11.2		24				RTOG 0631
	1	0.035		16		24				RTOG 0631
	1	5		8.8		8				
	1		50%	14.5		18,39,53	**2**		**16**	After 4–10 months, G3‐4 ulceration encountered
	1		50%	12.5		19,50	**2**		**16**	After 4–10 months, G3‐4 ulceration encountered
Bowel: Duodenum	1		50%	12.5		50	**1**		**77**	
	3	5		21		26,54,55,56				
	3	5		15		6,8				
	3				24	6,8,53				
	3				21	51				
	5				32	8				
	5	5		18.3		6				
	5	5		18		8				
	5				27.5	6				
	6	0.5		30		57				
	1	5		11.9		24				RTOG 0631
	1	0.035		15.4		24				RTOG 0631
	1				19	8				
	1	5		9.8		8				
	3	10		16.2		6				
	3	5		16.2		8				
Bowel: Ileum	3				27	6,8				
	5	10		19.5		6				
	5	5		19.5		8				
	5				35	8				
	5				29	6				
	1	5		11.9		24				RTOG 0631
Bowel: Jejunum	1	0.035		15.4		24				RTOG 0631
	1				19	8				
	1	5		9.8		8				
	3	10		16.2		6				
	3	5		16.2		8				
Bowel: Jejunum (cont'd.)	3				27.0	6,8				
	5	10		19.5		6				
	5	5		19.5		8				
	5				35	8				
	5				29	6				
	3	5		21		54,55,56				
Bowel: Large Intestine	3				24	53				
	6				30	48				
Bowel: Rectal Mucosa	4				28.5	37,38				
	4				41.8	37				minor deviation
Bowel: Rectal wall	4				38	37,38				
	1	20		14.3		24				RTOG 0631
	1	0.035		18.4		24				RTOG 0631
	1				22	8				
	1	20		11		8				
	2	1		14.25		35				RTOG 0321
	3				30	8				
	3	20		20.4		8				
	3				20	36				RTOG 9708, after 45Gy conventional
	4				33	39				
Bowel: Rectum	4				28.5	58				
	5				38	8				
	5	20		25		8				
	5	1		36		40,41				
	5		5%	36.25		59				
	5		10%	32.625		59				
	5		20%	29		59				
	5		50%	18.125		59				
	5				21	42,44				RTOG 0116 & 0118, after 45Gy conventional
	5				20.5	43				RTOG 0417, after 45Gy conventional
	1				12	53,60				
	3	5		21		54,55,56				
	3				30	61,62				
Bowel: Small Intestine	3				24	53				
	3				10	36				RTOG 9708, after 45Gy conventional
	6				30	48				
	6	0.5		30		57				
	1		5%	22.5		53				
	1		4%	22.5		50%	**4**		**77**	50% isodose line should not reach distal wall
	1		50%	14.5		53				
	1	10		11.2		24				RTOG 0631
	1	0.035		16		24				RTOG 0631
	1				16	8				
	1				12.4	25				RTOG 0915
	1	10		13		8				
	1	10		11.2		25				RTOG 0915
	1				12	53,60				
	3	5		21		26,54,55,56				
	3	10		15		6				
Bowel: Stomach	3				30	61,62,63				
	3				24	6,8,53				
	3	10		21		8				
	3				21	51				
	4				27.2	25				RTOG 0915
	4	10		17.6		25				RTOG 0915
	5				32	8				
	5	10		28		8				
	5	10		18.3		6				
	5				27.5	6				
	5	1		25		52				
	6				30	48				
	6	0.5		30		57				
Brachial Plexus: Ipsilat.	1	5		14.4		24				RTOG 0631, limit is for sacral plexus
	1	0.035		18		24				RTOG 0631, limit is for sacral plexus
	1	5		14		24				RTOG 0631
	1	0.035		17.5		24				RTOG 0631
	1				17.5	25				RTOG 0915
	1				16	8				Also for sacral plexus
	1	3		14.4		8				Also for sacral plexus
	1	3		14		25				RTOG 0915
	3				24	6,8,27,39,45, 46,53,64,65				RTOG 0618, Also for sacral plexus
Brachial Plexus: Ipsilat. (cont'd.)	3	3		22.5		8				Also for sacral plexus
	4				42.5	28	**1**		**27**	20% of br. plex. >40gy in one pt who had G3 AE
	4		20%	40		28	**1**		**27**	20% of br. plex. >40gy in one pt who had G3 AE
	4				40	27,28				Accuray STARS
	4	1		35		27,28				Accuray STARS
	4	10		30		27,28				Accuray STARS
	4				27.2	25				RTOG 0915
	4	3		23.6		25				RTOG 0915
	5	1		40		52				
	5				32	8,31				Also for sacral plexus, RTOG 0813, QOD
	5	3		30		8,31				Also for sacral plexus, RTOG 0813, QOD
Brain	5		100%	20		15				RTOG 7361 for palliative whole brain
	1				15	8				
	1	1		10		8				
	1				8	tradition				Preferred when possible
	1	0.5		7		tradition				Preferred when possible
Brain‐stem	3				23	8				
	3	1		18		8				
	5				31	8				
	5	1		26		8				
	5		100%	20		15				RTOG 7361 for palliative whole brain
	1	1		22.11		66	**1**	**1**	**114**	
	1				22	8				Limit is for ipsilat. bronchus
	1				20.2	25				RTOG 0915
Bronchi	1	4		10.5		25				RTOG 0915
	1	4		8.8		8				Limit is for ipsilat. bronchus
	3				30	6,8,27,39,46, 53,64,65				RTOG 0618, Limit is for ipsilat. bronchus
	3	5		21		54,55,67				
	3				20	68				Limit is for main bronchi
	3	4		15		8				Limit is for ipsilat. bronchus
	4				50	27				
	4	1		40		27				
	4	10		35		27,28				
Bronchi (cont'd.)	4				34.8	25				RTOG 0915
	4				31.6	29,30				
	4	4		15.6		25				RTOG 0915
	5				52.5	31				RTOG 0813, Limit is for ipsilat. bronchus, QOD
	5				45	69	**1**	**1**	**1**	Severe late bronchial effect
	5				38	8				Limit is for ipsilat. bronchus
	5	4		18		8,31				RTOG 0813, Limit is for ipsilat. bronchus, QOD
	1		5	14		24				RTOG 0631
	1	0.035		16		24				RTOG 0631
	1				17	70				
	1				16	8				
	1	5		14		8				
	1	2.9		8		70				
	1	1		8		34				
	1				15.6	71				Highest allowed value; a goal was not stated
Cauda Equina	3				24.2	7				Median doses quoted, not limits
	3				24	8				
	3	5		21.9		8				
	3	0.1		18.5		7				Median doses quoted, not limits
	3	1		10.7		7				Median doses quoted, not limits
	3	2		8.9		7				Median doses quoted, not limits
	3	5		6.2		7				Median doses quoted, not limits
	5				34	8				
	5	5		30		8				
	1				15	17	**7**	**9**	**50**	77.8% chance of RON above 15Gy
	1				13	72				
	1				12	73	**2**	**29**	**215**	7% chance of RON above this, 1.1% chance below
	1				11	74				
Chiasma	1				10	8,17,72,75	**4**	**15**	**50**	No RON below 10Gy, 27% RON from 10–15Gy
	1	0.2		8		8				
	1				8	72,73,76				
	3				19.5	8				
	3	0.2		15		8				
Chiasma (cont'd.)	5				25	8,72,77,78				
	5	0.2		20		8				
	5		100%	20		15				RTOG 7361 for palliative whole brain
	1				30	17				3rd, 4th, and 6th cranial nerves
Cranial Nerves	1				20	17				5th cranial nerve
	1				12	8				
Ears: Cochlea	3				20	8				
	5				27.5	8				
	1	5		11.9		24				RTOG 0631
	1	0.035		16		24				RTOG 0631
	1	2		20		66				Minor deviation
	1	1		24.31		66	**1**		**114**	
	1	1		22.88		66	**2**		**114**	Symptoms began 2 weeks after SBRT
	1	2		15		66				
	1				19	8				
	1				15.4	25				RTOG 0915
	1	5		14.5		8				
	1	5		11.9		25				RTOG 0915
	1				14	53,60				
	3	10		16.2		6				
Esophagus	3				27	6,8,27,39,45,				RTOG 0618
						46,53,64,65				
	3				24	53				
	3	5		21		8,26,67				
	3				20	68				
	4				50	27				
	4	1		35		27,28				
	4	10		30		27,28				
	4				30	25				RTOG 0915
	4	5		18.8		25				RTOG 0915
	4				20.8	29,30				
	5				52.5	31				RTOG 0813, QOD
	5				35	8				
	5	5		27.5		8,31				RTOG 0813, QOD, ‘non‐adjacent wall'
	5	10		19.5		6				
	5				29	6				
Esophagus (cont'd.)	5	1		25		52				
	6	0.5		30		57				
	1				3	39				
	1				2	79				
	2				6	39				
Eyes: Lens	2				3	39				Preferred cumulative max
	3				7	39				
	3				3	39				Preferred cumulative max
	5				7	39				
	5				3	39				Preferred cumulative max
	1				5	39				
	2				10	39				
	2				5	39				Preferred cumulative max
Eyes: Retina	3				15	39				
	3				5	39				Preferred cumulative max
	5				15	39				
	5				5	39				Preferred cumulative max
	1				22	8,24,25				RTOG 0631&0915, heart/pericardium
	1	15		16		8,24,25				RTOG 0631&0915, heart/pericardium
	3	15		24		8				
	3	5		21		26,54,55				
	3				30	6,8,27,39,46,				RTOG 0618
						53,61,64,65				
	3				20	68				
	4				50	27				
Heart	4	1		40		27,28				
	4	10		35		27,28				
	4				42.4	29,30				
	4				34	25				RTOG 0915, heart/pericardium
	4	15		28		25				RTOG 0915, heart/pericardium
	5				52.5	31				RTOG 0813, QOD, heart/pericardium
	5	1		40		52				
	5				38	8				
	5	15		32		8,31				RTOG 0813, QOD, heart/pericardium
	6				40	48,57				
Hilus	3				20	68				
	1	200		8.4		8,24				200cc must be spared, RTOG 0631 renal cortex
	1				2	39,80				Max dose to either kidney
Kidney: Comb.	3	200		14.4		8				200cc must be spared
	3	200		8.4		6				200cc must be spared
	5	200		17.5		8				200cc must be spared
	5	200		9.5		6				200cc must be spared
Kidney: Contra‐lat.	1		5%	5		18,39				
	1		50%	1.5		18,39				
	1		5%	5.8		18,39				
	1		50%	2		18,39				
Kidney: Ipsilat.	1		75%	5		50				75% of each kidney must be spared
	3	130		12.3		6				130cc must be spared
	3		33%	15		26,51,53,54,62,63				2/3 of kidney must be spared
	5	130		14.5		6				130cc must be spared
	1				5	39				
	2				10	39				
	2				5	39				Preferred cumulative max
Lacriminal Gland	3				15	39				
	3				5	39				Preferred cumulative max
	5				15	39				
	5				5	39				Preferred cumulative max
	1	4		10.5		24				RTOG 0631, also for trachea
Larynx	1	0.035		20.2		24				RTOG 0631, also for trachea
	5				20	49				QOD, re‐treat
	1	700		9.1		8				700cc of normal liver must be spared
	1		30%	12		53,60				
	1		50%	7		53,60				
Liver	1		50%	5		50				
	1		70%	2.5		50				70% of normal liver must be spared
	3	700		17.1		6,8				700cc of normal liver must be spared
	3	700		15		26,53,61,62,63				700cc of normal liver must be spared
	3		35%	15		62				35% of normal liver must be spared
	3		35%	24		53				
	3		33%	21		26,54,55,56				
	3		50%	20		51				
Liver (cont'd.)	3		50%	15		26,54,55,56				
	5	700		21		6,8				700cc of normal liver must be spared
	6		50%	22		48				Limit is for mean dose, but 50% means median
	6		50%	25		57				Limit is for mean dose, but 50% means median
	1		10%	20		66				
	1	1500		7		8,25				Must spare 1500cc of normal lung, RTOG 0915
	1	1000		7.4		8,24,25				Must spare 1000cc nrml lung, RTOG 0631&0915
	1		50%	2.5						Extrapolated from (28)
	2		50%	4						Extrapolated from (28)
	3		35%	15		45				
	3		15%	15		45	1		38	
	3		20%	20		27				May subtract GTV
	3		15%	20		39,53,64				RTOG 0618, Minor deviation
	3		10%	20		39,53,64				RTOG 0618
	3	1500		10.5		8				1500cc of normal lung must be spared
	3	1000		11.4		8				1000cc of normal lung must be spared
	3		10%	13		6				
	3		30%	10		27				May subtract GTV
Lungs	3		50%	12.5		81	5	9	128	50% risk of G2 RP, Extrapolated from their Table 4
	3		50%	8.5		81	4	28	128	14% risk of G2 RP, Extrapolated from their Table 4
	3		50%	8		6				
	3		50%	7		6				
	3		50%	6		81	**4**	**60**	**128**	7% risk of G2 RP, Extrapolated from their Table 4
	3		50%	5		27				May subtract GTV
	4		20%	20		27,28				May subtract GTV
	4		30%	10		27,28				May subtract GTV
	4	1500		11.6		25				Must spare 1500cc of normal lung, RTOG 0915
	4	1000		12.4		25				Must spare 1000cc of normal lung, RTOG 0915
	4		50%	14		81	**5**	**9**	**128**	50% risk of G2 RP, Extrapolated from their Table 4
	4		50%	9.4		81	**4**	**28**	**128**	14% risk of G2 RP, Extrapolated from their Table 4
	4		50%	6.5		81	**4**	**60**	**128**	7% risk of G2 RP, Extrapolated from their Table 4
	4		50%	5		27				May subtract GTV
	4		40%	5		28				May subtract GTV
	5	1500		12.5		8,31				Must spare 1500cc nrml lung, RTOG 0813, QOD
	5	1000		13.5		8,31				Must spare 1000cc nrml lung, RTOG 0813, QOD
	5		20%	25		52				
	5		20%	20						Copied from (27,28)
	5		30%	10						Copied from (27,28)
	5		8%	20		52	1		50	
Lungs (cont'd.)	5		11%	15		52	**1**		**50**	
	5		50%	2.36		52	**1**		**50**	Limit is for mean dose, but 50% means median
	5		50%	15		81	**5**	**9**	**128**	50% risk of G2 RP, Extrapolated from their Table 4
	5		50%	10		81	**4**	**28**	**128**	14% risk of G2 RP, Extrapolated from their Table 4
	5		50%	7		81	**4**	**60**	**128**	7% risk of G2 RP, Extrapolated from their Table 4
	5		20%	37.5		40				
Neurovas. Bundle	5		50%	38		41				
	1				15	17	**7**	**9**	**50**	77.8% chance of RON above 15Gy
	1				13	72				
	1				12	73	**2**	**29**	**215**	7% chance of RON above this, 1.1% chance below
	1				11	74				
	1				10	8,17,72,75	**4**	**15**	**50**	No RON below 10Gy, 27% RON from 10–15Gy
	1				8	8				tradition, dose tolerance not fully appreciated
	1	0.2		8		8,72,73,76				
	1				7.5	82				
	2	0.03		10		83				
	2				10	39				
	2				5	39				Preferred cumulative max
Optic nerve	3				19.5	8				
	3	0.03		15		83				
	3	0.2		15		8				
	3	0.5		10.5		83				
	3				15	39				
	3				5	39				Preferred cumulative max
	5				30	84				Only based on two cases
	5				25	8,72,77,78				
	5	0.03		25		83				
	5	0.2		20		8				
	5	0.05		20		83				
	5	0.5		12.5		83				
Penile Bulb	1				34	8				
	1	3		14		8				
	3				42	8				
	3	3		21.9		8				
Penile Bulb (cont'd.)	5				50	8				
	5		50%	29.5		40,41				
	5	3		30		8				
	1		33%	10.6		8,24				RTOG 0631
Renal Hilum	3		33%	18.6		8				
	5		33%	23		8				
	1	0.035		26		24				RTOG 0631
	1				26	25				RTOG 0915
	1				16	8				
	1	10		23		24,25				RTOG 0631&0915
	1	10		14.4		8				
	3				54	23	**3**		**50**	54gy 5mm deep, large‐scale necrosis G3‐4
	3				48	63	**1**		**21**	48gy 1cm deep, G3 fibrosis requiring intervention
	3	1		35		27				
	3	10		30		27				
Skin	3				30	45	**1**		**38**	30gy 1mm from surface
	3				24	8,64				RTOG 0618
	3				21	45				
	3	10		22.5		8				
	3				20	68				
	4	1		40		27,28				Surface to 5mm
	4	10		35		27,28	**3**		**27**	Surface to 5mm
	4				36	25				RTOG 0915
	4	10		33.2		25				RTOG 0915
	5				32	8,31				RTOG 0813, QOD
	5	10		30		8,31				RTOG 0813, QOD
	1		10%	10		7,24,85				10% of {cord adjacent to tumor +6 mm inf & sup}
	1		10%	9.6		85	1		86	Lower extremity G4/5 weakness
	1	0.9		8		80				
Spinal Cord	1	0.1		13.7		85,86	**1**		**86**	Lower extremity G4/5 weakness
	1				14.6	85,86	**1**		**86**	Lower extremity G4/5 weakness
	1				14	7,8,25,71				RTOG 0915
	1				13.1	86,87	**1**		**72**	Ipsilateral hemiplegia and contralateral pain
	1	0.1		6.9		86,87	**1**		**72**	Ipsilateral hemiplegia and contralateral pain
	1				13	7,70				
	1				10.6	86,87	**1**		**72**	After 5 months, classic Brown‐Sequard syndrome
	1	0.1		8.5		86,87	**1**		**72**	After 5 months, classic Brown‐Sequard syndrome
	1				10	7,88,89				
	1		100%	8		7				
	1		100%	10		7				
	1	0.35		10		24,25				RTOG 0631&0915
	1	0.25		10		8				
	1	0.2		10		90				
	1	2.6		8		70				
	1	1.7		8		91				
	1	1.2		7		8,24,25				RTOG 0631 SBRT only, RTOG 0915
	1	0.02		8		86,87	**3**		**72**	Ref (86) reports higher doses for 2 of these 3
	1				5	50				
	2				25.6	86,88	1			Bilateral leg weakness & urinary retention
	2	0.1		24.7		86,88	**1**			Bilateral leg weakness & urinary retention
Spinal Cord (cont'd.) 3				30.9	86,87,90	**1**		**55**		Posterior column dysfunction, motor weakness
	3	0.1		27.8		86,87,90	1		55	Posterior column dysfunction, motor weakness
	3	8		16.5		6				
	3	1.7		24		87,90	**1**		**55**	Posterior column dysfunction, motor weakness
	3				24	39				For ‘extreme cases’ only
	3				22	8				
	3				21	51,67				Based on BED3=45Gy, but BED1=55Gy
	3				18.6	7				Median doses quoted, not limits
	3	0.25		18		8				
	3	0.1		16.3		7				Median doses quoted, not limits
	3	1.2		11.1		8				
	3	1		8.5		7				Median doses quoted, not limits
	3	2		6.9		7				Median doses quoted, not limits
	3	5		4.1		7				Median doses quoted, not limits
	3				18	6,27,39,45,46				RTOG 0618
						53,62,63,64,65				
	3				15	26,54,68				
	4				26	25				RTOG 0915
	4				25	27				
	4	1		20		27,28				
	4	0.35		20.8		25				RTOG 0915
	4	1.2		13.6		25				RTOG 0915
	4	10		15		27,28				
	4				8.8	29,30				
	5				30	8,31				RTOG 0813, QOD
	5	1		25		52				
	5	8		20		6				
Spinal Cord (cont'd.)	5				24	92				
	5	0.25		22.5		8,31				RTOG 0813, QOD
	5	0.5		13.5		31				RTOG 0813, QOD
	5				22	6				
	5				20.4	7,93				Median max dose
	5	1.2		13.5		8				
	5				10	94,95				To allow for future reirradiation
	6				27	48,57				
	1	4		10.5		24				RTOG 0631, also for larynx
	1	0.035		20.2		24				RTOG 0631, also for larynx
	1				22	8				
	1				20.2	25				RTOG 0915
	1	4		10.5		25				RTOG 0915
	1	4		8.8		8				
	3				36	45				
	3	5		21		54,55,67				
3					6,8,27,39					RTOG 0618
Trachea					30	46,53,64,65				
	3	4		15		8				
	4				50	27				
	4	1		35		27,28				
	4	10		30		27,28				
	4				34.8	25				RTOG 0915
	4	4		15.6		25				RTOG 0915
	5				52.5	31				RTOG 0813, QOD
	5				38	8				
	5	4		18		8,31				RTOG 0813, QOD
Urethra	2	1		23.75		35				RTOG 0321
	4		10%	41.8		37				
	4				47.5	58				
	4				45.6	37,38				
Urethra (cont'd.)	4		50%	39.9		37				
	4				35	39				
	5		20%	47		41				

The second column in the table shows the number of fractions for each dose limit. Some of the tolerance limits are presented in terms of absolute volume in cubic centimeters (cc), while others are presented in terms of percentage of the total volume of the structure. For the absolute volume format, the third column of the table displays the specified volume in cc. Alternatively, for the relative volume format, the fourth column of the table presents the specified volume in percent. For either the absolute or relative volume constraints, the fifth column of the table shows the corresponding dose tolerance limit in Gy. In contrast to the volume‐dose limits, some of the dose limits are posed in terms of the maximum allowed point dose, and these are located in column six. No dose limit has an entry in both columns five and six; they are mutually exclusive. Column seven provides all references we found that mention the dose limit, and some brief notes are occasionally mentioned in the last column.

Columns eight through ten list adverse events that were reported in the literature. Column eight lists the number of adverse events (AE) reported at the dose level. The focus in this manuscript is Common Terminology Criteria for Adverse Events (CTCAE)^(16)^ Grade 3 events or higher, although some Grade 2 adverse events are also recorded in column eight. Column nine lists how many patients received the relevant dose range to the corresponding critical anatomical structure. Column ten lists how many patients were enrolled in the study. Columns eight and nine show that some of the dose tolerance limits presented in Table 1 are associated with a high percentage of AE and should be avoided.

Some of the dose tolerance limits for kidney, liver and lung are posed in terms of a critical volume instead of an allowed volume. This means that instead of allowing a certain small volume of a critical structure to exceed a particular dose, the requirement is that a certain critical volume of the structure must be spared, remaining below the specified dose. In the notes in Table 1 we write “must be spared” to denote these instances.

Some of the dose tolerance limits in Table 1 are for one to five fractions of high‐dose radiation following a course of conventionally fractionated radiation, such as the 23 Gy in five fraction bladder limit from RTOG 0417. In general, the topic of retreatment is beyond the scope of this manuscript, and most of this type of limit comes from high dose radiation (HDR) brachytherapy treatments rather than SBRT, but some researchers have used this conceptually to consider what could be achievable in a retreatment setting.

It must be noted that dose tolerance limits and dose‐volume histogram (DVH) analysis only show part of the overall situation. The location where the high dose occurred could sometimes be more important than how high the dose is. For example, with the steep dose gradients achievable with stereotactic delivery systems it would be possible to cover the entire cross‐sectional area of the spinal cord with a high dose, yet still only exceed the dose tolerance limit in one or two cc. In particular, the 8 cc spinal cord dose tolerance limits from Chang and Timmerman^(6)^ should be used with caution. Timmerman's more recent very thorough update^(8)^ now presents spinal cord volume‐dose limits in terms of 0.25 cc and 1.2 cc volumes, instead of the previous 8 cc volume. In general, circumferential irradiation of any critical structure should be avoided whenever possible.

## IV. DISCUSSION

The Emami limits are for 1/3, 2/3 and whole structure irradiation. In contrast, most of the SBRT limits are for the maximum point dose or a small percentage of the volume. Even though large volume limits are not explicitly stated, the entire premise for SBRT is based on small targets, and SBRT usually isn't used in cases where large volumes of critical structures would receive high dose. Therefore, in addition to the explicitly stated dose limits, SBRT clinicians need to ensure that large volumes of critical structures are not significantly exceeding conventional limits. Neglecting this constraint could result in high integral doses at ablative levels, with unknown consequences. Furthermore, small volume dose limits may be more affected by organ motion than large volume limits (e.g., the dose to 1 cc of bowel is more uncertain that the dose to 1/3 of bowel) – so integrated critical structure volumes may be advisable.

Several SBRT publications do present occurrences of Grade 5 adverse events. The CTCAE clearly categorizes Grade 5 as death for any type of adverse event.^(16)^ Compounding the problem, many authors don't report the actual doses that the failed critical structure received. The maximum point dose in a plan could be double the prescription dose – or at the other extreme, the failed critical structure could have received a dose much lower than the prescribed dose.

Few authors state how many patients actually reached dose limits and among those that do, care must be taken to interpret the probability estimates correctly. For example, in one study, seven patients out of fifty experienced radiation‐related optic neuropathy (RON) when they received doses of 15 Gy or higher to the chiasm or optic nerve in a single fraction.^(17)^ However, this does not imply a 7/50=14% chance of adverse event because the authors state that only nine patients had chiasm or optic nerve doses exceeding 15 Gy. Therefore, the actual estimate of adverse event should be 7/9=78%, which is significantly higher. The number of patients actually receiving the stated dose is among the most important validation of dose tolerance limits, but we only have an entry in column nine of Table 1 for 4% of the limits. We hope that future trials will document more clearly how many patients received each dose limit. Furthermore, when adverse events do occur, it would be most helpful if the involved critical structure doses would be published.

Some dose limits might result in toxicity that was not reported in the original reference. For example, the single fraction dose tolerance limit of “5% duodenum allowed to exceed 22.5 Gy” from Koong et al.^(18)^ is higher than the five fraction dose tolerance limit of “5 cc duodenum is allowed to exceed 18.3 Gy” from Chang et al.,^(6)^ even when radiobiological effects are not considered. Taking BED into account, assuming α/β=3, the Koong limit is about five times higher than the Chang limit. It is hard to imagine how such a higher limit could be employed without significant side effects, and yet Koong et al.^(18)^ state that, “at these doses, no Grade 3 or higher acute gastrointestinal toxicity was observed.” However, the Stanford group subsequently reported that two patients who were treated in accordance with their duodenal tolerance limits ultimately developed Grade 3–4 complications 8–10 months after treatment.^(^ ^18^ ^,^ ^19^ ^)^ This level of toxicity might be justified in attempts to aggressively attack a lethal disease such as pancreatic cancer that has few other options, but such high doses are probably not justified for more readily curable cases. Each physician must still carefully assess which dose limits are appropriate for each of their patients.

Historically, many dose tolerance limits were derived from computer simulations, animal studies, irradiated tissue samples in petri dishes and theoretical equations. None of these theoretically derived dose limits are directly presented in this manuscript. All the dose limits in this work have been previously published by various authors with apparently clinical intent. Several of the limits are from HDR brachytherapy, which typically is performed with significantly lower photon energy, although several authors have validated that some dose tolerance limits from HDR apply fairly well to SBRT. Gamma Knife delivery also uses photons with lower energy (about 1.25 MV) than most linear accelerator techniques (typically 6–18 MV), although many clinical users commonly interchange dose tolerance limits from one treatment modality to another.

Targeting accuracy can significantly affect the application of dose tolerance limits. Gamma Knife is still the gold standard in radiation targeting accuracy, so to apply Gamma Knife dose tolerance limits to treatments using conventional linear accelerators that have potentially much poorer accuracy could be precarious. The CyberKnife has a submillimeter end‐to‐end accuracy specification, so the historical Gamma Knife dose tolerance limits should have great relevance. However, it must be cautioned that the submillimeter specification is measured only with a phantom. There is currently no means to measure accuracy to that precision in a patient, and the non‐ideal geometry of each patient, in addition to organ movement and deformation, can potentially lead to larger‐than‐expected targeting errors if great expertise is not applied. To date, the CyberKnife is the only radiation therapy system available with live tracking of target motion – the CyberKnife actually moves with the target while the radiation beam is on.

Other technological improvements are able to improve the accuracy of conventional linear accelerators. Although the Novalis system (BrainLAB Inc., Munich, Germany) does not have a submillimeter end‐to‐end alignment specification, two research groups have shown that it is possible to achieve this.^(^ ^20^ ^,^ ^21^ ^)^ More recently, the SAlinac/submillimeter XKnife system has been demonstrated to achieve submillimeter end‐to‐end alignment accuracy on a conventional linac equipped with stereotactic cones.^(22)^

Dose tolerance limits will not have their intended benefit if the dose calculation is not accurate. For example, Hoppe et al.^(23)^ showed that the posterior skin dose could be 80% higher than expected if the immobilization devices or couch top creates a bolus effect. Clinicians might have a false sense of security if they are relying on inaccurate dose calculations.

## V. CONCLUSIONS

The majority of the initial SBRT patients had a relatively short life expectancy. This meant that short‐term efficacy and safety of the techniques could be quickly established. Extending life expectancy by even a few months could statistically be verified within a few years. In this frail patient population with many other comorbid conditions, short‐term adverse events would be seen fairly quickly. Therefore we would expect that with so many researchers using similar dose tolerance limits, many of these limits should be fairly accurate for short term effects.

Long‐term efficacy and safety is much more difficult to determine, especially given the nature of late effects and the relative short life expectancy of many of the patient populations studied. The late effects might not occur frequently in patients that often do not live that long – but that does not imply that the corresponding dose tolerance limits are safe for patients with a much longer life expectancy. This is an area of intense interest to many researchers and these dose tolerance limits may evolve significantly over the next few years. Hopefully this manuscript will be a useful reference in this endeavor.

## ACKNOWLEDGMENTS

This work is dedicated to the people who are persevering in the fight of their lives against cancer. The first author's mother was diagnosed with stage 4 lung cancer with pleural effusion and spinal metastasis and given six weeks to live. That was five years ago and she recently celebrated yet another birthday! While it is true that she has spent some time in the wheelchair and in hospitals and various clinics around the country for every therapy imaginable, she also spent much of those years attending family reunions, going on train rides and hiking with the grandkids, joining their birthday parties, going on walks with the ladies from the church, taking a cruise, and visiting relatives and friends in 12 states by plane, train and automobile – with a new appreciation of just what a precious gift from God every day of life is. When the doctors say “it's over” that doesn't always mean it's over! For any patient who is determined to continue the fight against cancer, we are committed to providing every reasonable option available, with the best level of precision, skill and knowledge humanly possible.
